# The influence of cell morphology on the dynamics and stability of model bacterial communities

**DOI:** 10.1371/journal.pcbi.1014874

**Published:** 2026-10-13

**Authors:** Ing Xhu Lim, Faisal Halabeya, Joshua N. Milstein

**Affiliations:** 1 Department of Chemical and Physical Sciences, University of Toronto Mississauga, Mississauga, Ontario, Canada; 2 Department of Physics, University of Toronto, Toronto, Ontario, Canada; Georg-August-Universitat Gottingen, GERMANY

## Abstract

Understanding how structures arise in microbial communities remains an outstanding challenge in bacterial ecology. When replicating within dense and confined environments, bacterial populations may collectively self-organize as a result of mechanical interactions. We previously observed that model dual-populations of bacteria, colonizing open-ended microchannels, either quickly fixate to a single population or segregate into long-lived, coexisting populations. This latter quasi-stability results from the alignment of bacteria into lanes, forcing cells to grow toward the open ends of the channel, with inter-lane invasion events driving the boundary dynamics between populations. Here we apply agent-based (AB) simulations to explore the boundary dynamics between dual-bacterial populations of varying morphology and division rate. We find that the simulated boundary dynamics are well-described by a simple drift-diffusion model, which enables us to estimate the mean time to fixation within competitive scenarios where the fixation time is difficult to access by AB simulations. Coccus-like cells display a competitive advantage over bacillus cells as they can more effectively invade and ultimately fixate, while coexisting populations of bacillus cells are “effectively” stable. And while faster-dividing cells should have a selective advantage, their morphology determines if this advantage drives fixation or acts as a defensive strategy to maintain their population. These findings highlight the critical role that cell morphology and mechanics play in shaping bacterial communities.

## Author summary

Bacteria are often found in multi-species communities, many of which are critical to human health, the environment, and industry. Within these communities, competition for resources shapes the overall population structure and resulting ecology. Model systems can provide a test-bed for understanding the interaction networks of these more complex communities. Here we show how mechanical forces between cells and their environment can be critical to the stability (or instability) of a bacterial community. Using simulations of dual-species competition within two-dimensional microchannels, along with mathematical modelling, we find that cell morphology can be a determining factor in the stability of a multi-species community.

## Introduction

Bacteria commonly exist within communities in nature where they compete for limited space and resources. These communities play an essential role in supporting a diversity of critical ecosystems, from regulating metabolism and protecting against pathogens within the human gut, to cycling carbon and nitrogen within Earth’s atmosphere [1,2]. Even initially well-mixed populations of bacteria will tend to segregate and spatially organize as cells grow and divide. This resulting spatial structure may be crucial for cooperation within species [3], the sharing of public goods such as digestive enzymes or metabolites [4], or as a resistance strategy toward antibiotic compounds [5,6]. While the availability of nutrients and the chemical environment both affect the spatial organization of bacterial communities, within dense systems, spatial structure may also emerge spontaneously through mechanical interactions with neighbouring cells and their confines [7,8]. This emergent behaviour may critically impact the evolution and ecology of these populations.

Mechanical interactions can affect both the invasion dynamics [9] and resulting self-organization [10] of bacterial communities. These effects are thought to give rise to much of the structural architecture of biofilms [11,12]. Collective mechanical forces may also directly influence the growth dynamics of bacteria [13], which may lead to to novel ecologies. For instance, while faster growing cells are often assumed to hold a selective advantage, within a number of dense and confined systems it has been observed that slower growing cells are able to either outcompete [14,15] or, at least, stably coexist with their faster growing peers [16].

Cellular morphology, which affects the packing of dense microcolonies, can also influence structural properties of these communities, as has been observed in the stratification of populations within 3-dimensional biofilms [17]. In simple confined monolayer systems, pill shaped *E. coli* cells will display a global nematic ordering arranging cells into distinct lanes [18]. The mechanisms behind this spontaneous ordering, as well as factors such as buckling instabilities which cause cells to invade neighbouring lanes and, ultimately, destroy such an arrangement, have been explored by numerous groups [19–22].

Synthetic microfluidic systems serve as a controlled test-bed for studying self-organized spatial structuring within bacterial communities [23,24]. We previously reported on the rapid emergence of quasi-stable, coexisting communities within a model competitive system composed of two distinct *E. coli* strains confined to open-ended, monolayer microchannels [16]. Cells seeded within these channels would either rapidly fixate to a homogeneous population of one cell type (typically in the time it takes for the populations to fill the channel, i.e., ~6−8 hrs), or would organize into distinct, quasi-stable populations. Once established, these segregated populations would coexist for extended times (> 24 hrs) despite significant heterogeneity between strains in terms of growth advantage or cell morphology.

These observations are largely explained by noting that, once the channel fills, the cells primarily self-align into lanes that grow toward the open ends of the channel. This arrangement is only altered by the rare cell invading into a neighboring lane and taking it over. We previously showed that successful invasions are, in fact, exceedingly rare for all attempts except those that occur in the middle of a lane, away from the open ends of the channel [25]. Most invading cells are quickly pushed out of the ends of the channel, which is why heterogeneous communities with a significant difference in growth rate between populations can coexist for extended periods.

Employing both agent-based (AB) simulations and mathematical modelling, we now explore the stability of these segregated communities by studying the dynamics of the boundary between the populations. In particular, we infer the mean first-passage time (MFPT) of the boundary to the channel edges, which indicates the average time it takes for the community to eventually fixate to a single, homogeneous population. The boundary dynamics are found to be well-modeled by a one-dimensional drift-diffusion equation whose parameters are influenced by the morphology and division rate of the bacteria.

Here we focus on competition between two bacterial strains displaying morphologies ranging from bacillus (pill-shaped) to coccus-like (round). These morphological differences can impart a selective advantage to the more coccus-like cells, such that the coccus-like population fixates within a finite period, whereas competition between increasingly elongated bacillus morphologies leads to mean fixation times that appear to diverge. And while an increased cell division rate can sometimes drive fixation, for well-aligned bacillus populations, it can at most stabilize the population by protecting it from invasion. Despite the simplicity of the system under study, our results highlight the role that bacterial morphology can play in the stability or instability of a bacterial community.

## Results

### Stability and boundary dynamics

We first employ agent-based (AB) simulations to model bacterial organization within dense communities that grow in two-dimensional, open-ended microchannels. AB simulations are a common tool for modelling microbial communities, and they are widely used to study population dynamics, community interactions, and emergent phenomena [26,27]. The AB simulations used here have been described in detail within a previous publication where they were shown to capture much of the behaviour observed in actual experiments [16]. Briefly, the mechanical interactions between bacteria are handled as a soft sphere model where the overlap of two soft spheres causes a repulsive Hertzian force. To mimic a monolayer where bacteria are confined, a damping coefficient is added to account for the medium and surface friction (see Methods).

While this previous work focused on understanding the relatively rapid dynamics of the populations either fixating to a single species or establishing segregated communities, here we focus on the longer-term dynamics of the latter, quasi-stable communities (states we will refer to as “coexistence”). All simulations are initialized with one cell of each of the two competitor strains randomly seeded within a microchannel (Fig 1A) where they are allowed to grow and divide (Fig 1B). We then ignore all populations that fixate to a single strain within 24 hrs (Fig 1C). The remaining systems will all have reached coexistence with the vast majority segregated into two populations separated by a boundary that runs along the long axis of the channel (Fig 1D). Bacillus cells will tend to organize into aligned lanes that span the channel, while more coccus-like cells will display a less ordered arrangement. Occasionally, we observe segregation into 3 distinct populations (< 10% of simulations) or into 4 or more populations (< 1% of simulations), but we will neglect these configurations in our initial analysis.

**Fig 1:**
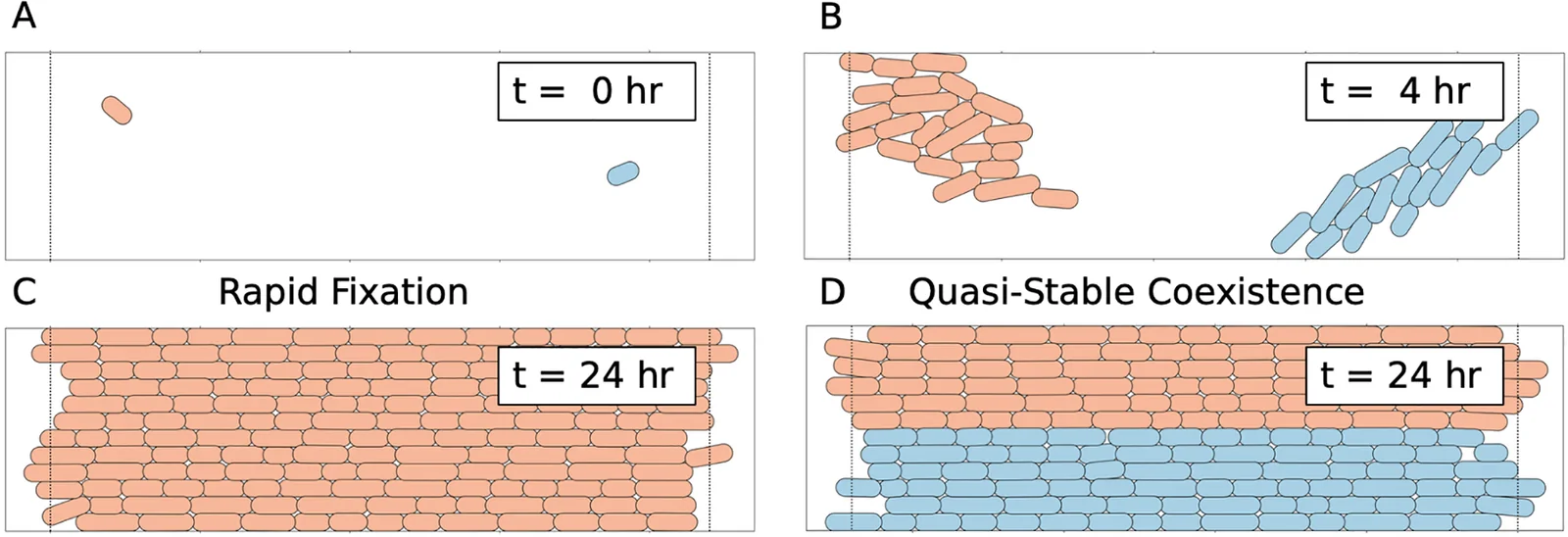
Rapid fixation versus quasi-stable coexistence. **A)** Cells are sparsely seeded within each channel with one cell of each strain randomly positioned and orientated. The dashed lines represent the open ends of the channel; once a cell crosses this line, it is removed from the simulation. **B)** Cells then spread out within the chamber due to multiple rounds of cell division. This will eventually lead to either **C)** fixation of a single strain or **D)** a quasi-stable, coexisting state where different populations, each composed of a single strain organized into lanes, segregate within the chamber. Cell division now primarily drives growth toward the open ends of the chamber.

We simulated competition between populations of cells with morphologies ranging from bacillus (pill-shaped) to coccus-like (round). The cells were modelled as rectangular segments of finite length *l* and 1 μm width with half-circular end caps 1 μm in diameter (see S1 Fig). This results in an initial length *L* of *l* + 1 μm that, for convenience, will be indicated by $L_{l+1}$. By increasing *l*, we move from coccus-*l*ike, to increasingly elongated bacillus cells. As an examp*l*e, Fig 2A illustrates competition between coccus-like cells and bacillus cells (*L*_1.0_ and *L*_2.5_, respectively). Figure 2B and 2C display the rapid fixation of the systems to either a pure bacillus or coccus-like population, respectively, while Fig 2D displays the comparatively slower dynamics of the two populations within the coexistence state. Throughout, we will employ the fractional area, which is the total area of a population divided by the area of the microchannel, as a convenient measure of the population abundance. Note, however, that due to edge effects at the channel boundaries, this number can fluctuate above 1.

**Fig 2:**
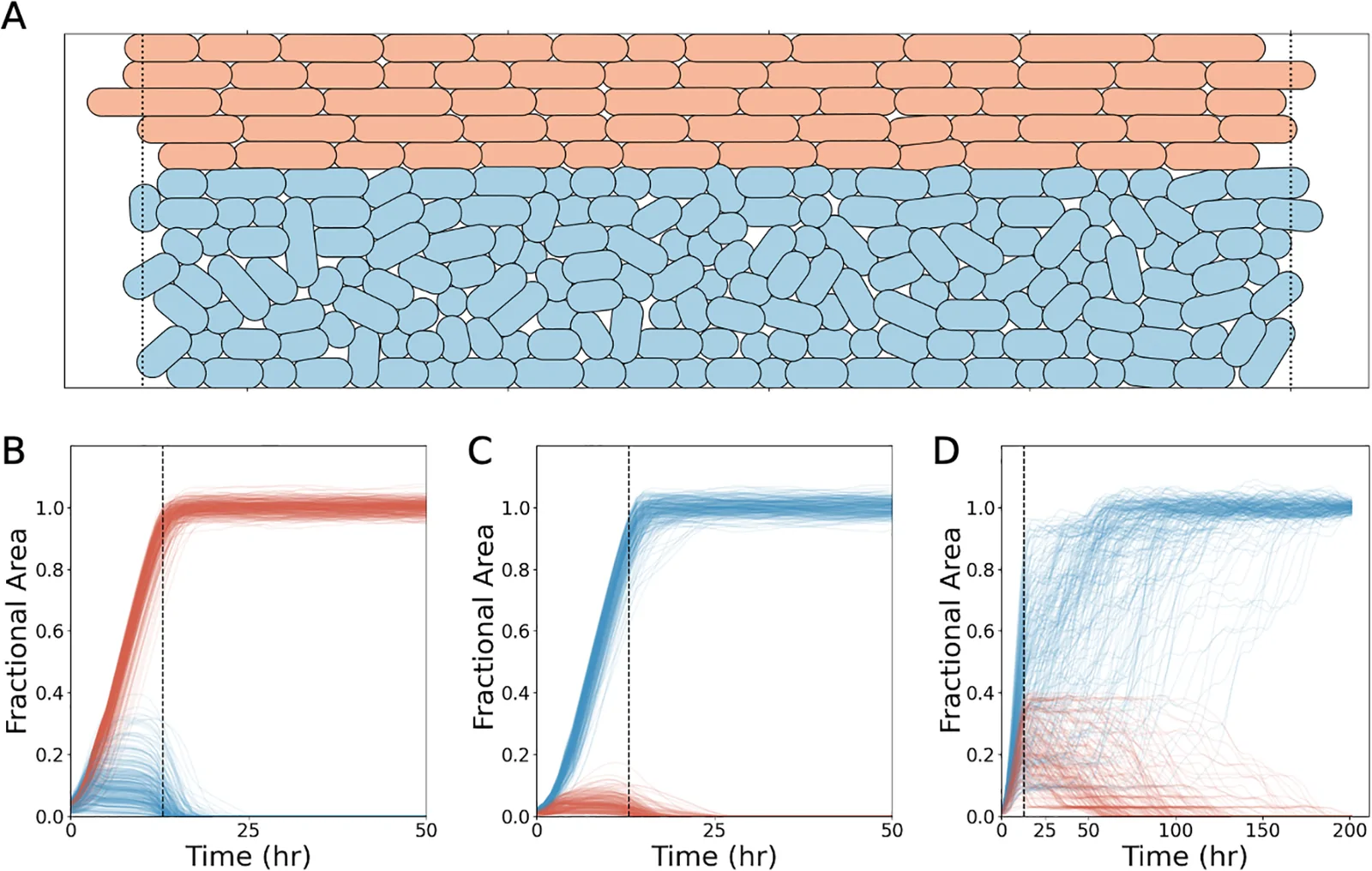
Fast versus slow fixation of dual competitive populations. **A)** During coexistence, the boundary between segregated populations evolves through repeated invasion attempts by cells at the boundary. Coccus-like cells (blue), with less of a propensity to self-align, are more likely to push into the domain of bacillus cells (red). We can separate the competitive outcomes into **B)** and **C)** fast (< 24 hrs) and **D)** slow (> 24 hrs) fixation events. Fast fixations occur when one population out-competes the other in an effort to fill the chamber, while slow fixations result from the boundary dynamics between segregated populations. For this example, the bacillus cells never out-compete the coccus-like cells once coexistence is reached.

Here we are primarily interested in the dynamical process of fixation from the coexistence state, which is driven by the motion of the boundary separating the two populations. To illustrate this, Fig 3A displays a kymograph of the relative position of the boundary over time showing a series of discrete jumps that correspond to successful invasions into one or more lanes of the neighbouring bacillus population. These discrete jumps are also observable in a plot of the fractional area versus time. The takeover of the bacillus cells by the coccus-like cells occurs in a predictable way as illustrated in Fig 3B. First, coccus-like cells invade into the neighbouring lane of bacillus cells with successful invasions almost always occurring near the centre of the chamber. Then, as the invading cells divide, their progeny slowly push the bacillus cells towards the open ends of the chamber until all bacillus cells in that lane have been expelled. These steps repeat, interspersed with many unsuccessful invasion attempts, until the simulation ends or the coccus-like cells take over the entire chamber.

**Fig 3:**
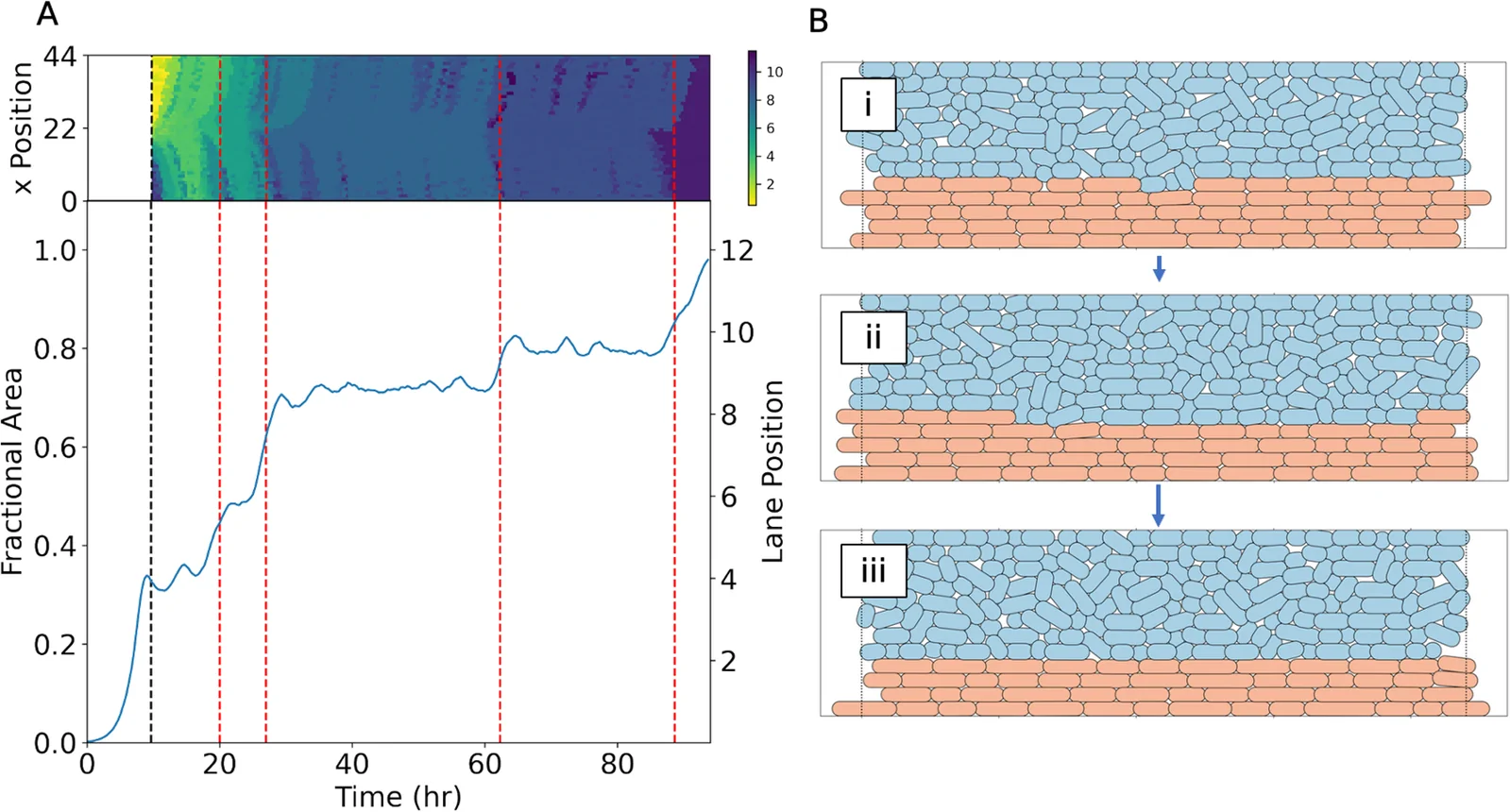
Boundary dynamics between competitive populations. **A)** Boundary dynamics from simulations of bacillus versus coccus-like cells (2.5 μm and 1.0 μm initial lengths, respectively). Top panel: Kymograph of the boundary dynamics as a function of time. The colours represent the spatial location of the boundary discretized into lanes within a channel supporting up to 12 lanes. Bottom panel: Fractional area with change-point analysis identifying successful invasions (red-dashed vertical lines) showing the boundary move in discrete jumps. The black-dashed vertical line indicates the chamber fill time. **B)** Boundary dynamics are driven by invasions. Illustrated here: i) coccus-like cells invade a lane of bacillus cells near the chamber centre, ii) cell division by the invaders then pushes the competing bacillus cells toward the edges of the chamber, and iii) the lane fixates to coccus-like cells shifting the location of the boundary downward. The dashed vertical lines indicate the chamber boundaries.

Since the timescale to fixation of a single lane, post invasion, is typically much faster than the translational motion of the boundary (Fig 3B), we may ignore the intra-lane fixation dynamics and approximate the boundary as moving in discrete jumps between lanes. Within this approximation, as we will show, the translational dynamics of the boundary may be modelled as simple drift-diffusive motion. By employing AB simulations to extract both the diffusion constants and drift velocities, for boundaries between cells of varying morphology, we first show that the model predictions agree well with AB simulations of systems that reach fixation from coexistence within about a week (corresponding, roughly, with the actual run-time of the simulations). However, our analytical model also enables us to predict the time to fixation for competitive systems when the fixation time becomes extremely long, making it difficult to assess through AB simulations alone.

### Drift and diffusion of the population boundary

The translational motion of the population boundary can be described by the Fokker-Planck equation, which characterizes the spatial and temporal evolution of the probability density function *P*(*x*,*t*) for the boundary:


$$\begin{array}{c} \frac{\partial P(x,t)}{\partial t}=-\mu \frac{\partial }{\partial x}\left[P(x,t)\right]+D\frac{\partial^{2}}{\partial x^{2}}\left[P(x,t)\right], \end{array}$$

where *t* is time, *x* is a spatial coordinate indicating the location of the boundary between the channel walls, μ represents the drift velocity, and *D* is a diffusion constant. We assume that both μ and *D* are constants, which should be a reasonable approximation for sufficiently wide channels (see S5 Fig and S3 Table). From repeated runs of the AB simulations, estimates of these parameters can be inferred from the simulated data using the following relationships:


$$\begin{array}{c} \langle \Delta x(t)\rangle =\mu t, \end{array}$$

$$\begin{array}{c} \sigma_{\Delta x}^{2}(t)=\langle \Delta x(t{)}^{2}\rangle -\langle \Delta x(t)\rangle^{2}=2Dt, \end{array}$$

where Δx is the relative displacement from the boundary’s initial position:


$$\begin{array}{c} \Delta x(t)=x(t)-x(0). \end{array}$$

### Effects of relative morphology and division time

We considered AB simulations of two bacterial populations with one bacillus population held fixed at an initial cell length of *L*_2.5_ and the other varied from *L*_1.0_ (coccus-like) to *L*_2.5_, as well as between two coccus-like populations each of initial length *L*_1.0_. For convenience, we will refer to the length ratio $L_{X}/L_{Y}$ to indicate the various competitive scenarios. Cells grew to double their initial length and divided at equal rates of one division every $t_{d}=60\pm 6$ minutes. Gaussian noise (10%) was introduced in the division rate to prevent synchronization of cell size and growth stage (see Methods).

Figure 4A and 4B display the observed dynamics of the relative displacement of the boundary Δx(t) for the first 40 hrs post-coexistence for the cases: $L_{1.0}/L_{2.5}$ and $L_{1.0}/L_{1.0}$. Once again, the relative area is used as a measure of the boundary coordinate *x*(*t*). A linear fit to the mean of the trajectories yields the drift velocity μ. Similarly, the insets show *t*he evolution of the variance $\sigma_{\Delta x}^{2}(t)$ for the first 40 hrs post-coexistence, which can be fitted linearly to extract the diffusion constant *D*.

**Fig 4:**
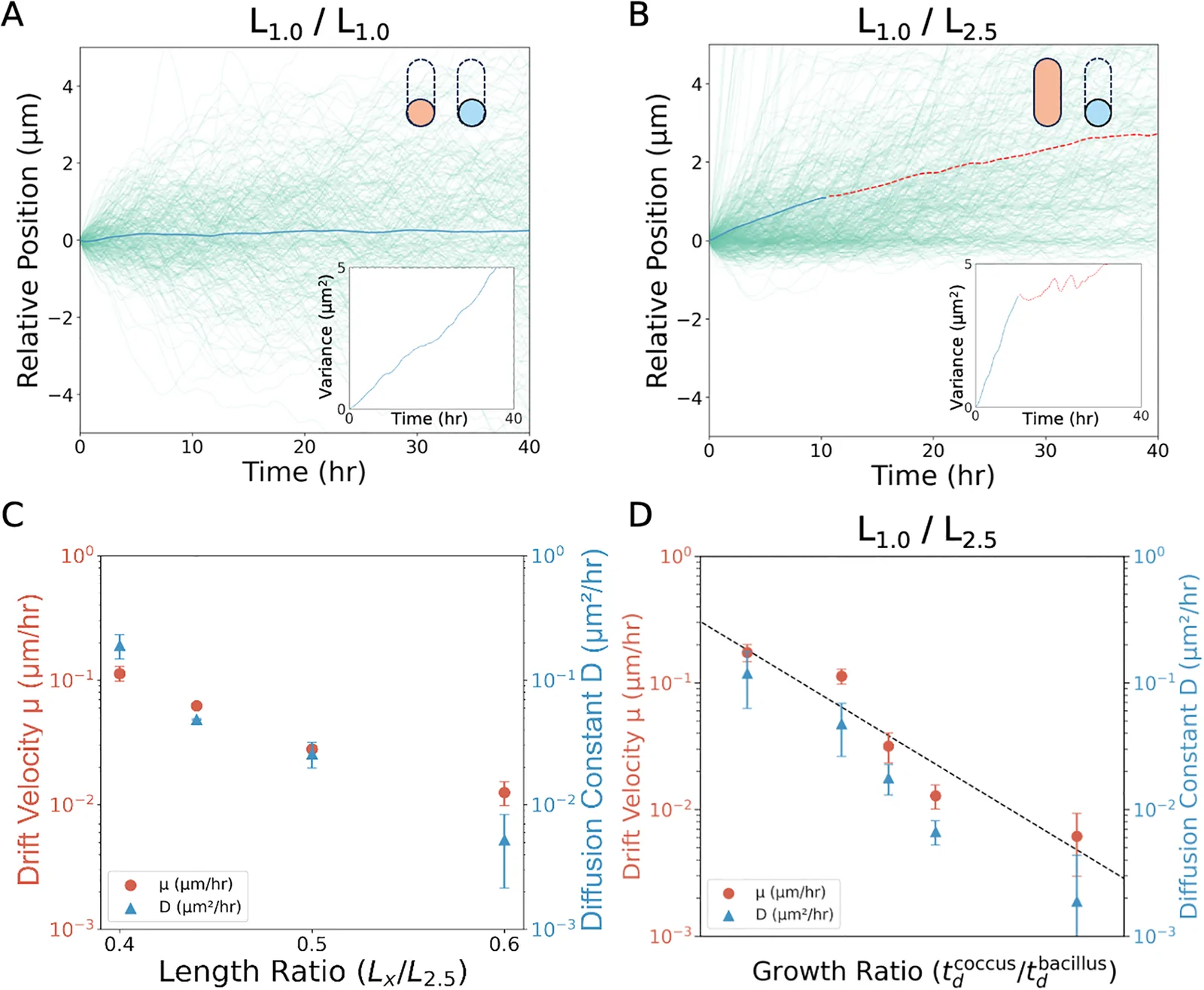
Drift-diffusive motion of the population boundary. Relative displacement of the population boundary as a function of time for competition between populations with the following morphologies (defined by the initial cell length ratio $L_{X}/L_{Y}$): A) $L_{1.0}/L_{1.0}$ and B) $L_{1.0}/L_{2.5}$. Shown are the simulated trajectories of the boundary (green lines) as well as the fitted mean of the trajectories (blue line). We exclude the data from the fit once a significant number of simulations fixate (red dashed line). Insets display the corresponding variance over time. Linear regressions quantify: (i) drift velocity (mean slope) and (ii) diffusion coefficient (variance slope). **(C)** Drift velocities (red) and diffusion coefficients (blue) as a function of morphology. Data points shown are for length ratios $L_{X}/L_{2.5}=$0.6, 0.5, 0.44, and 0.4. **(D)** Drift velocities (red) and diffusion coefficients (blue) as a function of growth ratio (i.e., $t_{d}^{coccus}/t_{d}^{bacillus}$ with $t_{d}^{coccus}$ fixed). $L_{1.0}/L_{2.5}$ competition (growth ratios of 0.5, 0.8, 0.9, 1.0, and 1.2). The dashed line indicates a linear fit to the log-log plot with slope ~−8. Each data point in C and D was obtained from 1000 simulations, as were the graphs in A and B.

Figure 4C summarizes the drift velocities (μ) and diffusion constants (*D*) extracted from simulations as a function of relative morphology (i.e., length ratios $L_{X}/L_{2.5}$), but only for those cases where we observed a significant number of lane changes, thus allowing us to reliably extract the diffusive parameters (see S2 Fig and S1 Table). Morphology is seen to provide a strong competitive advantage, with the drift velocity–-which largely determines the dynamics–-increasing by orders of magnitude as bacillus cells compete with increasingly coccus-like cells. Under these conditions, with the drift always promoting invasion into the bacillus population, the more coccus-like cells dominantly fixate (see S4 Fig). Conversely, any dynamics between comparable bacilli populations is exceedingly slow, effectively jamming the two populations into a coexisting state. For competition between dual coccus-like populations, as expected, the dynamics of the boundary are largely diffusive (for $L_{1.0}/L_{1.0}$, the drift and diffusion constants were 0.0066±0.0084 $\mu_{m}/hr$ and 0.0328±0.0030 $\mu m^{2}/hr$, respectively). Note, very little dynamics were observed for more elongated, neutral systems, which is why we only present the results for $L_{1.0}/L_{1.0}$. Similar trends are observed if we maintain one strain as coccus-like and vary the morphology of its competitor by elongating those cells (see S7 Fig and S4 Table).

We next considered the effects of varying the relative division time between populations. We analyzed AB simulations of competition between elongated bacillus and coccus-like cells ($L_{1.0}/L_{2.5}$) keeping the mean division time of the coccus-like populations constant (60±6 minutes) while ramping up the division time of the bacillus cells and maintaining Gaussian noise of ±10% about the mean. In Fig 4D, we display the resulting drift-diffusion parameters as we decreased the relative division time (i.e., growth ratio $t_{d}^{coccus}/t_{d}^{bacillus}$) by a factor of 2 (see S3 Fig and S2 Table).

While the drift velocity of the boundary decreased by orders of magnitude as the division time of the bacillus population doubled, the drift remained into the bacillus population, meaning that the coccus-like cells were still the population which dominantly fixated. A faster division rate, while not contributing to an increase in self-fixation, instead protected the bacillus cells for longer from colonization and eventual fixation by the coccus-like population. Coccus-like cells, attempting to colonize the bacillus population, are instead rapidly pushed to the boundary and ejected from the population before they are able to take over a new lane. In our simulations, we observed an exponential dependence of the drift of the coccus-like population as a function of division time (exponent α=−8), which illustrates how the rate of cell division can significantly alter population dynamics.

### Mean first-passage times

For one-dimensional diffusion, where both the drift μ and diffusion *D* are constant, we can derive a second-order differential equation for the mean first-passage time τ(q) [28],


$$\begin{array}{c} \left[\mu \frac{\partial }{\partial q}+D\frac{\partial^{2}}{\partial q^{2}}\right]\tau (q)=-1. \end{array}$$ (1)

where 0≤q≤W specifies the starting point between the two walls of the microchannel. For absorbing boundary conditions such that τ(0)=τ(W)=0, this can be solved to yield the rescaled mean first-passage time (MFPT):


$$\begin{array}{c} \tau (z)=\frac{W}{\mu }\left[\frac{e^{-\gamma z}-1}{e^{-\gamma }-1}-z\right]. \end{array}$$ (2)

Here γ=μW/D and we scale the starting position of the boundary to the chamber width *z* = *q*/*W*. In the limit of zero drift (μ→0) this becomes


$$\begin{array}{c} \tau_{0}(z)=\frac{W^{2}}{2D}z\left(1-z\right). \end{array}$$ (3)

Employing the diffusive parameters found from our AB simulations, we may use Eq. 2 to predict the MFPTs for the various competitive scenarios shown in Fig 4.

To validate the derived MFPT model, theoretical predictions were compared with the empirical data obtained from simulations for $L_{1.0}/L_{2.5}$ competition. For this scenario, all populations fully fixated from coexistence within the maximum simulated time of 200 hrs. This happens to roughly correspond to the simulation run time, or a little over a week on our cluster. A comparison of the fixation times displayed in simulations and MFPT predictions, for possible initial positions of the population boundary, is shown (inset Fig 5A).

**Fig 5:**
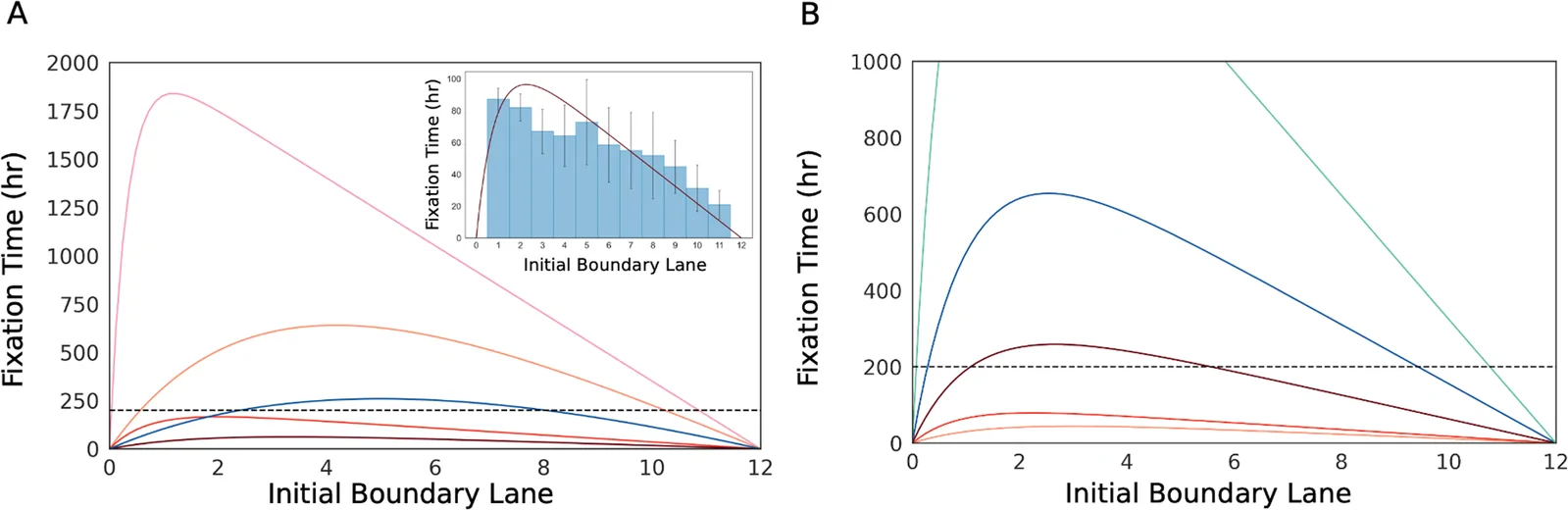
Theoretical predictions of fixation times for population boundaries that initialize at varying positions between the channel walls. This is shown **A)** as a function of length ratio (top to bottom: $L_{X}/L_{2.5}=$ 0.6, 0.5, 0.44, 0.4) and **B)** as a function of growth ratio for fixed $t_{d}^{bacillus}$ (top to bottom: $t_{d}^{coccus}/t_{d}^{bacillus}=$ 0.5, 0.8, 0.9, 1.0, 1.2) under $L_{1.0}/L_{2.5}$ competition. A simulation cutoff time of 200 hrs is indicated (black horizontal dashed line). The inset in (A) displays the average fixation times for $L_{1.0}/L_{2.5}$ competition at a growth ratio of 1. Bars represent the average fixation times at each initial boundary position, with the curve indicating the predicted MFPTs.

Most of the simulations, at less distinct relative morphologies, did not fixate a sufficient number of times during the simulation to extract the full MFPT distribution. For these instances, using the drift-diffusion parameters from the previous section, we simply employ our MFPT model to predict the fixation times. Figure 5A shows the predicted fixation times as a function of initial boundary position for competition between length ratios of $L_{X}/L_{2.5}=$ 0.6, 0.5, 0.44, and 0.4. As we vary the length of the initially coccus-like population toward that of the *L*_2.5_ bacillus competitor cells, the predicted fixation times rapidly increase, leading to an increasingly stable coexistence of the two populations. Likewise, the predicted fixation times for $L_{1.0}/L_{2.5}$ competition, when varying the relative division time between populations, are shown in Fig 5B. As the division time of the bacillus population is decreased, the time to fixation of the coccus-like population, from coexistence, is increasingly extended.

## Discussion

In this study, employing agent-based simulations and mathematical modelling, we investigated the role of bacterial morphology in determining the stability of spatially structured microbial communities confined within a 2D monolayer. Within these dual-population systems, cells tend to spatially order into distinct lanes of a given cell type with competition driven by successful inter-lane invasions.

We find that rounder, more coccus-like cells consistently display an advantage over elongated, pill-shaped bacillus cells due to an increased affinity for invading neighbouring lanes, which more effectively drives the system toward fixation. This competitive advantage can be mitigated by increasing the growth rate of the bacillus cells. However, doing so does not promote fixation of the bacillus population; rather, it only protects the population from invasion and eventual fixation by the coccus-like competitor strain.

A simple one-dimensional drift-diffusion model for the boundary between populations provides an estimate for the long-term fixation dynamics. Here we employ this model to predict the fixation time for systems whose boundary dynamics become exceedingly slow, such that the populations form a jammed or quasi-stable equilibrium, which is the case for competition between similarly elongated bacillus populations. These slow dynamics are difficult to access through AB simulations alone.

One could argue that more sophisticated algorithms than those we employed might provide access to longer time scales. Still, we have shown that both morphology and division dynamics can greatly stabilize these mixed communities. The question remains, however, as to whether these competitive systems are ever truly stable such that the fixation time approaches infinity.

Likewise, while it should be possible to extract the drift and diffusion constants for these systems from a microscopic model alone, here we have relied on phenomenological AB simulations to infer these dynamical values. Indeed, when we vary the parameters in our AB simulations, these values quantitatively change. For instance, if we hinder the rotation of cells upon interaction with neighbouring cells, the boundaries are less dynamic and the systems tend to take longer to fixate (see S6 Fig and S3 Table). However, we do not expect these effects to change our qualitative observations or the trends we have discussed so much as the absolute values of the fixation times. More realistic AB simulations, that incorporate the microscopic interactions between cells and the environment, and which go beyond our simple soft-sphere hydrodynamic model, may provide further insight into these dynamical systems.

Collectively, our findings highlight the influence of cell morphology on the stability of competing bacterial communities. Morphological traits, through purely mechanical interactions, can strongly influence the long-term competitive outcomes and ecological trajectories of bacterial populations. Morphology can give rise to dynamic and spontaneous structure within bacterial communities, which can affect competitive dynamics in unexpected ways. Within these structures, seemingly obvious advantages–-such as being able to replicate faster–-may not always lead to the dominance of a population. Similarly, phenotypic traits such as cell morphology, in certain settings, may play a role in eradicating persistent populations resistant to other forms of treatment. These findings deepen our understanding of microbial ecology, provide valuable insight into how microbial communities form collective structure, and may lead to more predictive tools for exploring and managing bacterial community dynamics across natural and engineered ecosystems.

## Methods

The AB model was primarily used to simulate a 2D rectangular environment with dimensions of 44 μm × 12 μm where cells are grown within a monolayer. The bacterial cells are modeled as agents of spherocylindrical shape (although, technically, only 2D) that grow to a maximal length at a given growth rate. Cells grow by exponentially increasing the length of the rectangular portion of the sphero-cylinder until the total length of the cell is twice the initial length, at which point the cell is split into two equal sized new sphero-cylinders (see S1 Fig). Physical contact between the cells happens when cells overlap with each other causing a repulsive Hertzian force (i.e., a soft sphere model) within a confined, damped environment. A damping coefficient ξ is is added to account for the liquid medium and surface friction from the confinement. This results in a set of equations for the translational and rotational motion of each agent (bacteria) within our simulation.

The full set of equations and specific parameters implemented in our AB model are provided in Ma et al. [16]. Each cell persists in the simulation until its centre exits the microchannel boundaries through either of the open ends. At this point, the cell is deemed to have exited the channel and is consequently eliminated from the simulation. In Ma et al. [16], the maximal length was drawn from a normal distribution of final lengths centered around twice the initial length. Here, we instead fix the final length; however, to make the cells asynchronous, we instead draw the growth rates from a normal distribution with a standard deviation of 10% of the cells growth rate around the defined growth rate. The agent-based model ignores the directional flow of the medium, which is consistent with other simulations in the field.

## Supporting information

S1 Fig Illustration of cell growth and division within the AB model. (JPG)

S2 FigBoundary dynamics for varying morphologies ($L_{x}/L_{2.5}$).Relative Position and Variance (inset) of the population boundary as a function of time for competition between populations with the following morphologies (defined by the initial cell lengths). Shown in (A-F) are $L_{X}/L_{2.5}$ for $L_{X}=$ 2.5 μm, 2.0 μm, 1.25 μm, 1.75 μm, 1.5 μm, and 1.1 μm, respectively. Shown are the simulated trajectories of the boundary (green lines) as well as the mean of the trajectories (blue line). Also indicated is where the data is no longer fitted due to too many simulations terminating, thus reducing sampling size (red dashed line).(JPG)

S3 FigBoundary dynamics for varying growth ratios.Relative Position and Variance (inset) of the population boundary as a function of time for competition between $L_{1.0}/L_{2.5}$ populations with the following growth ratio (defined as $t_{d}^{dcoccus}/t_{d}^{bacillus}$ with $t_{d}^{coccus}$ fixed). Shown in (A-D) at growth ratios of 1.2, 0.9, 08, and 0.5, respectively. Shown are the simulated trajectories of the boundary (green lines) as well as the mean of the trajectories (blue line).(JPG)

S4 FigFixation time distributions and cumulative fixation probability.Fixation time distribution across simulations of A) L_1.0_ / L_2.5_, B) L_1.1_ / L_2.5_, C) L_1.25_ / L_2.5_. Fixation time is taken as the time it takes for the simulation to fixate after post 24 hr. D) Cumulative probability distribution for fixation as a function of time. The L_1.0_ / L_2.5_ simulations all reach fixation within the 200 hr simulation run time. In contrast, the L_1.1_ / L_2.5_ and L_1.25_ / L_2.5_ conditions only showed fixation for trajectories that began very close to a fixation state. This explains the plateau in the figure. Because the lane positions are discrete, the next available initial lane positions have a mean first passage time much longer than 200 hr. The orange lines indicate the theoretical prediction for drift-diffusive motion and is given by the relationship $\sum_{i}\frac{a_{i}}{\sqrt{2\pi \sigma^{2}t^{3}}}\exp\left(-\frac{(a_{i}-\mu t{)}^{2}}{2\sigma^{2}t}\right)$. Here μ is the drift, *D* is the diffusion constant, $\sigma^{2}=2D$, and $a_{i}$ is the distance between the boundary and the chamber wall taken from the initial condition of simulation *i*. Since we only observe fixation by the more coccus cells in the simulations shown, here we employ the PDF for drift-diffusion with one absorbing boundary.(JPG)

S5 FigBoundary dynamics for varying channel widths.Relative position and variance (inset) of the population boundary as a function of time for competition between populations with varying channel widths. A), B), and C) depict simulations of $L_{1.0}/L_{2.5}$ competition while D), E), and F), depict simulations of $L_{1.0}/L_{1.0}$ competition. In A) and D) the width of the chamber has been increased to allow for 18 lanes (18 μm wide), for B) and E) 12 lanes (12 μm wide), and for C) and F) only 6 lanes (6 μm wide).(JPG)

S6 FigBoundary displacement for increased moment of inertia.Relative position and variance (inset) of the population boundary as a function of time for competition between populations with restricted rotational motion. We artificially affected the rotational motion of cells by doubling their moment of inertia, which leads to less rotational motion of the cells. The simulation shown is for $L_{1.0}/L_{2.5}$ competition.(JPG)

S7 FigBoundary dynamics for alternate morphologies ($L_{x}/L_{1.0}$).Relative displacement of the population boundary as a function of time for competition between $L_{1.0}/L_{x}$ populations where one population is held at a constant cocci-like morphology. A), B), and C) depict simulations of $L_{1.0}/L_{1.0}$, $L_{1.0}/L_{1.5}$, and $L_{1.0}/L_{2.5}$ competition respectively.(JPG)

S1 TableDrift and diffusion constants for varying morphologies.Table of drift and diffusion constants from S2 Fig. The errors in our estimates of the drift constants are found by first calculating $y_{i}\pm \sigma_{i}$, where $y_{i}$ is the mean and $\sigma_{i}$ is the standard deviation of the relative position data at each time point *i*. Points at $y_{i}+\sigma_{i}$ and $y_{i}-\sigma_{i}$ are each linearly fitted to generate two lines. We then take the difference between these two slopes as the error in the drift constant. Error in our estimation of the diffusion constant was simply derived by bootstrapping at a size of 80% of the original data.(JPG)

S2 Table Drift and diffusion constants for varying growth ratios. Table of drift and diffusion constants for S3 Fig. The errors from drift and diffusion were calculated in the same manner as previously.
(JPG)

S3 Table Drift and diffusion constants for varying channel widths and increased moment of inertia. Table of drift and diffusion constants extracted from S5 and S6 Figs. The errors from drift and diffusion are taken in the same manner as previously described.
(JPG)

S4 Table Drift and diffusion constants for alternate morphologies. Table of drift and diffusion values from S7 Fig. The errors from drift and diffusion are taken in the same manner as previously described.
(JPG)
