# Supplementary material for: The influence of cell morphology on the dynamics and stability of model bacterial communities
Source: PLoS Comput Biol. 2026 Oct 13;22(10):e1014874. doi: 10.1371/journal.pcbi.1014874 (PMC13674858; doi:10.1371/journal.pcbi.1014874)
Supplement: Supplementary file 1 [file pcbi.1014874.s014.pdf]

9/17/26

Dear Editor,

We are pleased to see that our revision addressed all concerns of Reviewer #1. We hope with this follow up submission that we will be able to alleviate any remaining concerns of Reviewer #2. For clarity, we reproduce the comments provided by the reviewer and indicate our response below.

1. "Thank you for adding the new 6 and 18-lanes setups. Looking at table S3, I am not sure it makes sense to call the 12-lanes and 18-lanes parameters nearly identical and the 6-lanes parameters very different. Comparing 12 and 18 lanes, the parameters are barely within error margins of each other. Also, 6 and 12 lanes is a much larger relative difference in system width than 12 and 18... Overall, based on this evidence, I would recommend the authors not to commit definitively to whether the parameters are width dependent or not (which should not be a problem since, as they point out themselves, their work is about the validity of the conceptual description, not about quantitative values)."

This is a valid critique. Within this revision we do not comment upon the width dependence, but our data remains available in the supplementary section for the interested reader. We maintain within the text only the following statement: "We assume that both μ and D are constants, which should be a reasonable approximation for sufficiently wide channels." Again, this does not say anything about the width dependence, just clarifies our assumptions.

2. "Related to this parameter change and possible edge effects in the 6-lanes setup, the authors indicated that "We have included a short discussion of this in the manuscript", but I couldn't find this change in the marked-up version of the manuscript (except the small comment that constant parameters seem to valid for large systems in line 149 of the marked-up version, which doesn't address/explain the new results very much)."

This was a mistake as the discussion indicated somehow did not make it to the final resubmission. We apologize for the confusion. However, in light of the previous comment, we would like to leave out any further discussion of the width dependence.

3. "Similarly, for the reduced-moment-of-inertia results, has there been a mixup of numbers in Table S3? The drift seems to be identical for 12 lanes with the regular moment of inertia and I/2. The two diffusion coefficients seem to be nearly within error margins, but more importantly, the one for I/2 seems to be even *lower*, so why do the authors interpret this as a "more dynamic" boundary? Some more explanation is needed. I am sorry if I am

missing something simple here… By the way, the table says L1/L2.5 while the title of Fig S6 says L1/L1.5 (different length ratio).”

Thank you for catching these errors. We have updated the tables and manuscript with our corrections. In fact, we should have been presenting additional simulations increasing the moment of inertia for all cells by a factor of 2. From the updated table, it’s clear that both the drift and diffusion of the boundaries increase when moving from 2I to I, which will lead to faster fixations. This is what we originally intended to indicate. Since we are doubling the moment of inertia compared to the case considered throughout the text, this is now noted in the text as follows: “For instance, if we hinder the rotation of cells upon interaction with neighbouring cells, the boundaries are less dynamic and the systems tend to take longer to fixate.” We then refer the reader to the Supp. Mat. We apologize for the confusion.

We also corrected the typo mentioned in the figure title.

4. “I still think the inset in Fig. 4C is difficult to interpret. Its main purpose currently seems to be (now that the error bar on the drift is larger) to show that there is some diffusion but no significant drift for the L1-vs-L1 case. To make this point, it would be easier/clearer to just state the values for drift and diffusion in the text where the inset is currently referenced, especially given that this case is now included in the new L1-vs-Lx data in table S4. One could either remove the inset in Fig. 4C or modify it to show all the data from Table S4 (in any case, it would be good to show the data from table S4 also as a plot). I assume the neutral case is not shown for the Lx-vs-L2.5 data because of the long time scales involved in the L2.5-vs-L2.5 competition? Maybe it would be good to (re)state explicitly that this is why this case was only analysed in the L1-vs-Lx data when talking about neutral competition.”

From the reviewer’s feedback, we felt it made the most sense to simply remove the insert. The reviewer is correct that the L1/L1 case is presented because, unlike the other cases shown where the motion of the boundary is largely dominated by the drift term, here there is no significant drift. For L2.5/L2.5, the boundary hardly moves and we never see fixations over the timescales we explored.

The text has been adjusted as follows (translated from LaTex):

“For competition between dual coccus-like populations, as expected, the dynamics of the boundary are largely diffusive (for L1.0/L1.0, the drift and diffusion constants were 0.0066 +/- 0.0084 um/hr and 0.0328 +/- 0.0030 um^2/hr, respectively). Note, very little dynamics were observed for more elongated, neutral systems, which is why we only present the

results for L1.0/L1.0."

5. "The added fixation time distributions in Fig. S4 are nice, but they don't really contribute to the raised point about model validation, as long as no theory prediction from the fitted model is included. So, overall, no additional model-vs-data comparison was performed. Perhaps the authors could comment in the discussion why such comparisons beyond the mean first passage time in the inset of Fig. 5A were not done (in my naive thinking, one could just plot a theory prediction for the first passage time distribution and compare that to the distribution of the fixation times in Fig. S4, but maybe I am overlooking something), or why certain things can be expected to match the model but others not."

We have provided the additional theoretical comparison suggested by the reviewer. We have added theoretical curves to Figs. S4 A-C for what would be predicted for drift-diffusive motion for the distributions presented. The drift and diffusion constants, as well as the initial positions of the boundaries, are all extracted from the respective simulations. This is discussed in the caption.

6. "A minor point: I would not call the inset in Fig. 5A a "histogram of fixation times". This would imply a distribution/count of some kind (as in Fig. S4), whereas the inset in Fig. 5A really just shows fixation time averages (and the authors happened to choose a bar plot)."

The caption has been edited to indicate that "The inset in (A) displays the average fixation times [...]".
