# Supplementary material for: The influence of cell morphology on the dynamics and stability of model bacterial communities
Source: PLoS Comput Biol. 2026 Oct 13;22(10):e1014874. doi: 10.1371/journal.pcbi.1014874 (PMC13674858; doi:10.1371/journal.pcbi.1014874)
Supplement: Supplementary file 2 [file pcbi.1014874.s013.docx]

Dear Editor,

We would like to thank both you and the reviewers for your time and effort in providing feedback on our manuscript *“The influence of cell morphology on the dynamics and stability of model bacterial communities.”* We have revised the manuscript to incorporate these comments, focusing on better contextualizing our work within the current literature, and exploring the generality of our results. Beyond modifications to the manuscript, we now include a Methods section along with significant supporting data and analysis from additional simulations within a greatly expanded Supplementary Information section. We provide our responses to each of the points raised by the reviewers below.

Reviewer #1

“The AB simulation described in a previous paper (Ma et al PNAS 2024) contains a few parameters that might affect drift and diffusion, notably the randomization parameter for angular orientations of daughter cells. Can the authors show, for a selected combination of morphologies, how this parameter affects the experiment? And can they include an argument why they choose a particular parameter in the current simulation?”

In the current simulations, there is no randomization of angular orientations, but the range of orientations is affected by the torque induced when cells collide. We have added a Methods section providing more details of our AB simulations for better clarity along with an additional figure in the Supplementary Information (S1 Fig.)

Still, to the reviewer’s point, within the Supplementary Information, we now provide data on the effects to the drift and diffusion parameters when we affect the rotational motion of the cells through their moment of inertia, which does indeed alter these parameters. Here we artificially reduce the moment of inertia of the cells by a factor of 2 allowing them to rotate more freely (S6 Fig. and S3 Table). We also discuss this in the text:

“Indeed, when we vary the parameters in our AB simulations, these values quantitatively change. For instance, if we allow the cells to more freely rotate upon interaction with neighbouring cells, the boundaries are more dynamic and the systems tend to fixate faster (see S6 Fig. and S3 Table).”

Again, our AB simulations are phenomenological, and the underlying parameters were chosen based largely on literature cited in Ma et al. PNAS 2024, and because these parameters match much of the behavior we have seen in our experiments as described in that paper. Here we are not necessarily concerned with the quantitative or absolute values for the drift and diffusion constants or fixation times; rather, we are more interested in the qualitative behavior or trends observed within these systems when one incorporates a minimal model for the cells bumping into one another. Of course, more realistic microscopic models that consider additional physics to model the interactions (such as cells bending or indenting) might add to the story we tell here. We have added a brief discussion to that effect in the Conclusion.

Reviewer #2

1. “If I understand correctly, the manuscript analyzes essentially one channel geometry supporting up to 12 lanes. This immediately raises the question what is meant to be universal here. Is the drift-diffusion description intended as a local effective model for interactions between neighboring columns, with coefficients that should be largely independent of the global channel width? Or are the inferred parameters expected to depend on the total number of lanes and thus on the global mechanical state of the system? Related statements in the paper, such as the distinction between fast and slow fixation or the possibility of subdivision into more than two domains, are also likely to depend on system width. The broader literature on proliferating rods in confinement (see point 4 below) suggests that ordering, buckling, and stress organization can be geometry-dependent, so some explicit exploration of width dependence, even at a modest level, would substantially strengthen the paper.”

We have added data to the Supplementary Information on the effects of changing the channel width (S5 Fig. and S3 Table), exploring a 6-lane and an 18-lane channel, and comparing the results to those from our original 12-lane system. We see that the drift and diffusion constants between the 12- and 18-lane systems are nearly identical, providing further evidence that we may approximate the drift and diffusion as constants. However, for the 6-lane system, the drift constant does change, indicating that for narrow enough channels, edge effects start to become significant. We have included a short discussion of this in the manuscript. Note that for the 18-lane system, as expected, we start to get a significant number of coexistence states composed of multiple boundaries. Since we are limiting the scope of this manuscript to studying the dynamics of a single boundary, we have not considered the multi-boundary equilibria that result from seeding these channels.

2. “At present, the reduction to a drift-diffusion description remains largely phenomenological. However, in this class of systems there is already a substantial body of literature connecting orientational order, its breakdown, and the underlying mechanics of confined proliferating rods. In channels and related confined geometries, nematic ordering has been linked to stress anisotropy and geometric confinement, while free and weakly confined colonies exhibit microdomain formation and (dis)ordering through the interplay of steric alignment and growth-induced extensile stresses. More recent work has further explored the role of strain-rate and subtle particle-shape effects for the stability of ordered states (see point 4). This raises the question how the effective lane-invasion dynamics described here are related to these previously identified mechanical ingredients (buckling, stresses, etc.). Even if the authors cannot address all of these points quantitatively within the present manuscript, they should at least discuss them explicitly and, ideally, probe some aspects directly.”

In the present manuscript, we have indeed employed a drift-diffusion equation as a phenomenological model of the motion of the barrier. A fully mechanistic model to describe either the boundary motion or the drift and diffusion parameters is beyond the scope of our present work. Still, as mentioned, we have explored how the drift and diffusion parameters are affected by other factors beyond what we presented previously. In particular, we now consider the effects of varying the channel width (S5 Fig. and S3 Table)), the angular motion of bacteria (S6 Fig. and S3 Table), and the absolute cell length of competitive populations (S7 Fig and S4 Table).

3. “I am not sure that plotting the results as a function of a length ratio is the most natural representation. For growth-rate effects, a ratio is plausible because one indeed expects relative exponential growth to be the key control parameter. For cell geometry, that is less obvious. The current x-axis of Fig. 4C suggests that only the ratio matters, but do the authors really expect the absolute initial lengths to not matter? Considering the importance of alignment, steric torques, and packing, this seems unlikely to me. Maybe it would be clearer to show the actual absolute lengths on one axis, or add explicit tests where one population is fixed at 1.0 µm and the other is varied. The inset for the neutral 1.0 µm versus 1.0 µm case is also hard to compare quantitatively with anything else (the data point for 0.4 in the main panel is already an additional "vs. 1.0µm" case, correct?). Moreover, I was surprised that the inferred drift for the nominally neutral 1.0 µm versus 1.0 µm case still appears appreciable and with relatively small error bars; I would have expected zero drift to lie within the error bars if positive and negative drifts are accounted for correctly.”

We did not intend to imply that the ratio of cell lengths is universal to the behavior we see. We have since changed our notation to specify the lengths of the two competing cells (L_X_/L_Y_), which should help clarify the figures and inserts. As for the neutral case, we have corrected a mistake in calculating the error bars, which are now appreciable and reflect our difficulty in extracting a value for drift since it sits around zero.

We have also included data (S7 Fig. and S4 Table) from a number of simulations where we maintain a cocci-like cell and extend the length of its competitor (L_X_/L_1.0_, where x runs from 1.0-2.5). Previously, we maintained a bacilli cell and shortened the length of the competitor (L_X_/L_2.5_, where x runs from 2.5-1.0). This is referenced and commented on within the text. We hope these additional simulations address the reviewers’ concerns.

4. “The relevant literature is currently a bit underrepresented/narrow, both on competition in spatially structured populations and on mechanically driven ordering in proliferating bacterial systems. The manuscript would benefit from being placed more carefully in the broader context of the field, showing that competition outcomes in dense microbial populations are shaped by mechanics, confinement, and geometry across a range of systems. Some (relatively random, but all not-yet-cited) examples include Karita et al., PNAS, 2022, in confined crypt-like chambers; Postek et al., PNAS, 2024, also on drift and selection channels; Coyte et al., PNAS, 2017, on spatial structure in porous environments; Nadell et al., eLife, 2017, on spatial competition in Pseudomonas aeruginosa biofilms; Giometto et al., PNAS, 2018, on physical interactions shaping selection; van den Berg et al., Nat. Comm., 2025 on the role of nematic alignment at expanding fronts. As background for complementary systems/mechanisms, there is also Hallatschek et al., PNAS, 2007, on genetic drift and sector formation at expanding frontiers, and Farrell et al., Journal of the Royal Society Interface, 2017, on how cell shape, friction, and colony mechanics affect surfing probabilities. Effective models have also been a topic in some of these publications. Of course it is up to the authors to decide what they want to include/highlight, but if overlaps/interactions/distinctions with respect to the literature were discussed in more detail (what does the discovered mechanism add/contribute?), this would also sharpen the focus of the manuscript.

At the same time, the paper also crucially relies on mechanically induced orientational order and its destabilization in monolayers of growing spherocylinders, and that side of the field should definitely be more thoroughly discussed. Relevant are, for example, Volfson et al., PNAS, 2008 on ordering; Boyer et al., Physical Biology, 2011 on the buckling instability; You et al., Physical Review X, 2018 on the geometry and mechanics of nematic microdomains; You et al., Science Advances, 2021 on confinement-induced self-organization; Isensee et al., Journal of the Royal Society Interface, 2022 specifically on the perfect ordering in confined populations of growing rods that is the basis for this study; Langeslay and Juarez, Soft Matter, 2024 on strain-rate-controlled alignment; and Isensee and Bittihn, Soft Matter, 2025 on the sensitive dependence on particle shape. This is of course a subjective selection and some might be more relevant than others, but I think mentioning/discussing some literature more thoroughly would be very helpful to provide both background and concrete mechanistic connections (see also points 1 and 2).”

We have expanded the Introduction significantly within this revision to better contextualize our work within the field. We now include a discussion on the role of mechanics in spatial ordering and its possibly counterintuitive effects on community ecology. We have also included a discussion on the role of cell morphology in spatial ordering. Most relevant to this manuscript, we focus on the alignment of cells into lanes and effects that lead to the modification or destruction of this ordering.

5. “The drift-diffusion reduction is validated only rather lightly. On the model building side, it is not shown how well the fits used to extract drift and diffusion parameters actually fit the data (and whether deviations are simply finite size effects or physically meaningful). Fixation time predictions are directly compared with theory only for one specific parameter set, while slower cases are extrapolated from fitted coefficients. That is not unreasonable given the long simulations required, but then the paper should be more careful in how strongly it states that the model “works.” There are several additional quantities that could be compared without requiring prohibitively long simulations: fixation probability within a fixed observation window, the distribution rather than only the mean of fixation times, the probability that the “wrong” population fixates when drift is small, etc. Some deeper validation would be very helpful, and if discrepancies emerge, that would actually improve the paper by clarifying the limits of the effective description.”

We now provide within the SI tables listing all drift and diffusion constants as well as the error in these estimates (S1-S4 Table). The figures (S2-S3 Fig. and S5-S7 Fig.) show additional details, such as the range over which these fits were performed. In addition, we provide data for cases not shown within the manuscript, such as when both cells are sufficiently bacilli that we see very few lane changes and thus cannot calculate the dynamical parameters. Likewise, as discussed above, we provide data on how the drift and diffusion constants change as the width of the channel increases to better validate our assumption that these parameters are spatially independent constants.

As suggested, to enhance our discussion, we also provide in S4 Fig. the fixation time distributions and cumulative fixation probability curves for a few illustrative simulations.

Finally, we have tried to mute the language with regards to the model since we cannot validate it for very long fixation times. For instance, in the Conclusion, instead of saying that the drift/diffusion model “effectively captures the long-term fixation dynamics” we now say that it “provides an estimate for the long-term fixation dynamics”.

6. “The authors refer readers to previous work and supporting information for the AB model, but given that the central claims concern mechanical mechanisms, the main text should briefly state the essential ingredients: Overdamped dynamics, steric force law, friction/mobility model, etc.. Without that, it is hard to judge which aspects of the conclusions are robust and which may depend on modeling details, a question which should also at least be discussed in the manuscript (for example, is more successful lane invasion of shorter cells exclusively a geometric phenomenon, or does relative mobility also play a role? That might depend on how these mobilities are chosen.).”

We have inserted additional text when we introduce the AB simulations to better clarify the essentials of our model. We also refer the reader to a deeper discussion within the Methods section (along with S1 Fig.).

As already mentioned, there are a number of places throughout the text where we discuss how altering the parameters within the AB simulation might alter the absolute values we extract from the simulations. While we have not explored the full range of parameter space within these simulations, as mentioned, we now provide simulations to show these effects when we vary the channel widths, alter the angular motion of bacteria, and tune the absolute cell length.

Minor comments:

1. Line 53: since the shapes differ mainly by length rather than width, “elongated” seems more suitable than “extended.”

“Elongated” fits the description more aptly. “Extended” has been changed to “elongated”.

2. Line 81: the geometry appears inconsistent as written; the end caps seem to have a *diameter* of 1 µm, not a radius of 1 µm, if they are meant to match a 1 µm-wide rectangular body.

We now refer to diameter instead of radius.

3. Line 146: to eliminate ambiguity, “promoting invasion *into* the bacillus population” would read more clearly.

Changed to “the drift always promoting invasion into the bacillus population”.

4. While it is mentioned in the model description that cell lengths are given in terms of their initial length just after division, it would help to repeat this in the relevant figure captions, since different conventions exist in the literature.

All figures mentioning lengths now specify that the lengths referenced are initial lengths. This is also clarified in the text where necessary.

5. Fig. 4C: the information that one length is kept constant at 2.5 µm while the other is varied is missing from the plot/caption and currently only appears in the text.

This is corrected with our new notation employed throughout of L_x_/L_2.5_.

6. Fig. 4D: the caption says “size ratios,” but should probably be growth-rate ratios.

Thank you for catching this error. Changed to “growth ratio” instead of size ratio.

7. Throughout the manuscript, the use of the word “coccus” is somewhat unusual and potentially misleading. In the model, all particles appear to be rodlike or capsule-like objects with varying aspect ratio; the shortest ones are not actual cocci in the biological sense (which could potentially go through different geometries during elongation/division). In many places it would be clearer to write “shorter cells,” “lower-aspect-ratio cells,” or “cells with shorter initial/division length.” instead of using "coccus" as an adjective like "more coccus", etc. ("coccus-like" seems fine to me).

All previous references to “coccus” cells are now changed to “coccus-like” cells.

8. A few small typographical issues I noticed:

* line 67 there appears to be a stray quotation mark in “dynamics of‘ the latter”;

Removed the stray quotation mark.

* line 77 has “less than < 1 %” is redundant;

Removed “less than”.

* line 177, usage of "U", while the drift parameter is denoted by μ elsewhere;

Changed to μ.

* in the caption for Fig. S2, “Each *are* taken from 1000 simulations”.

Changed to “Each configuration is sampled from 1000 simulations”.
