# Supplementary material for: The influence of cell morphology on the dynamics and stability of model bacterial communities
Source: PLoS Comput Biol. 2026 Oct 13;22(10):e1014874. doi: 10.1371/journal.pcbi.1014874 (PMC13674858; doi:10.1371/journal.pcbi.1014874)
Supplement: Peer review history

# Peer review: Decision Letter 0

**DOI:** 10.1371/journal.pcbi.1014874.r001

**Authors:** Stefan Klumpp, Tobias Bollenbach

30 Mar 2026

The influence of cell morphology on the dynamics and stability of model bacterial communities

PLOS Computational Biology

Dear Dr. Milstein,

Thank you for submitting your manuscript to PLOS Computational Biology. After careful consideration, we feel that it has merit but does not fully meet PLOS Computational Biology's publication criteria as it currently stands. Therefore, we invite you to submit a revised version of the manuscript that addresses the points raised during the review process.

Please submit your revised manuscript by May 30 2026 11:59PM. If you will need more time than this to complete your revisions, please reply to this message or contact the journal office at ploscompbiol@plos.org. When you're ready to submit your revision, log on to https://www.editorialmanager.com/pcompbiol/ and select the 'Submissions Needing Revision' folder to locate your manuscript file.

Please include the following items when submitting your revised manuscript:

* A letter that responds to each point raised by the editor and reviewer(s). You should upload this letter as a separate file labeled 'Response to Reviewers'. This file does not need to include responses to formatting updates and technical items listed in the 'Journal Requirements' section below.

* A marked-up copy of your manuscript that highlights changes made to the original version. You should upload this as a separate file labeled 'Revised Manuscript with Track Changes'.

* An unmarked version of your revised paper without tracked changes. You should upload this as a separate file labeled 'Manuscript'.

If you would like to make changes to your financial disclosure, competing interests statement, or data availability statement, please make these updates within the submission form at the time of resubmission. Guidelines for resubmitting your figure files are available below the reviewer comments at the end of this letter

We look forward to receiving your revised manuscript.

Kind regards,

Stefan Klumpp

Academic Editor

PLOS Computational Biology

Tobias Bollenbach

Section Editor

PLOS Computational Biology

**Additional Editor Comments:**

While both reviewers appreciate the approach taken here and acknowledge its usefulness, they also point to some weaknesses of the manuscript that you should try to alleviate. In particular the questions how general the results are (e.g., geometry-dependence) and the relation to the extensive existing literature need to be addressed. This does not mean that you have to do everything proposed by the reviewers or to cite all references the reviewer mentions, but the work should be put into context since that much relevant literature already exists and also addresses some of the questions that are left open here.

**Journal Requirements:**

If the reviewer comments include a recommendation to cite specific previously published works, please review and evaluate these publications to determine whether they are relevant and should be cited. There is no requirement to cite these works unless the editor has indicated otherwise.

1) Please ensure that the CRediT author contributions listed for every co-author are completed accurately and in full.

At this stage, the following Authors/Authors require contributions: Faisal Halabeya, Ing Xhu Lim, and Joshua N. Milstein. Please ensure that the full contributions of each author are acknowledged in the "Add/Edit/Remove Authors" section of our submission form.

The list of CRediT author contributions may be found here: https://journals.plos.org/ploscompbiol/s/authorship#loc-author-contributions

2) We ask that a manuscript source file is provided at Revision. Please upload your manuscript file as a .doc, .docx, .rtf or .tex. If you are providing a .tex file, please upload it under the item type u2018LaTeX Source Fileu2019 and leave your .pdf version as the item type u2018Manuscriptu2019.

3) Your manuscript is missing the following sections: Results, Discussion, and Methods.  Please ensure all required sections are present and in the correct order. Make sure section heading levels are clearly indicated in the manuscript text, and limit sub-sections to 3 heading levels. An outline of the required sections can be consulted in our submission guidelines here:

https://journals.plos.org/ploscompbiol/s/submission-guidelines#loc-parts-of-a-submission

4) Please upload all main figures as separate Figure files in .tif or .eps format. For more information about how to convert and format your figure files please see our guidelines:

https://journals.plos.org/ploscompbiol/s/figures

**Reviewers' comments:**

Reviewer's Responses to Questions

**Comments to the Authors:**

**Please note here if the review is uploaded as an attachment.**

Reviewer #1: The work by Xhu Lim et al studies the influence of bacterial cell morphology and growth rate on the long-term competition between strains in wide 2D channels that are open on both ends. This particular confining geometry has been used by the same authors to study short-term dynamics of bacterial competition in previous works, both experimentally and theoretically. Here, they use agent-based simulations to study time scales not easily accessible experimentally. They previously demonstrated that rod-shaped bacteria tend to form lanes in the confining geometry that stabilizes their coexistence. Here, they demonstrate that rod-shaped cells are displaced by coccoid cells at long times, and they also show that increasing growth of one population increases its lifetime in the channel. Simulation results are coarse-grained using a one-dimensional drift-diffusion model that is then validated in terms of first-passage (population extinction) times.

Together this is a well written manuscript. All the conclusions are well demonstrated.

I only have one minor comment:

The AB simulation described in a previous paper (Ma et al PNAS 2024) contains a few parameters that might affect drift and diffusion, notably the randomization parameter for angular orientations of daughter cells. Can the authors show, for a selected combination of morphologies, how this parameter affects the experiment? And can they include an argument why they choose a particular parameter in the current simulation?

Reviewer #2: The manuscript by Lim et al. investigates two-strain competition of rod-shaped cells with different lengths in open-ended monolayer microchannels using agent-based simulations and an effective stochastic description of the boundary between competing populations. After an initial transient, many realizations enter a long-lived coexistence regime in which cells segregate into lanes, and eventual fixation proceeds through rare successful invasions of neighboring lanes. The authors coarse-grain this process into a one-dimensional drift-diffusion model for the population boundary, infer drift and diffusion coefficients from simulation data, and use mean-first-passage theory to estimate fixation times. Their main conclusion is that shorter, more rounded cells invade more effectively than elongated cells, whereas increasing the growth rate of the elongated population mainly acts defensively by slowing invasion rather than reversing the fixation bias. The manuscript is clearly written and the reduction to an effective boundary dynamics is potentially useful.

The topic is timely and relevant. There is substantial current interest in how cell shape, growth, steric interactions, confinement, and mechanics shape ordering, stresses, buckling, and competition in dense microbial populations. In that sense, this is in principle a nice and well-written paper. However, the scientific substance presently feels rather lightweight. The analysis is demonstrated for one fairly specific setup, and the generality of the proposed effective description remains unclear. The manuscript would also benefit from being placed more carefully in the context of both the mechanical-ordering literature and the literature on competition/fixation in spatially structured proliferating populations. These connections are currently underdeveloped and are important for interpreting the results. See below for details.

Major points:

1. If I understand correctly, the manuscript analyzes essentially one channel geometry supporting up to 12 lanes. This immediately raises the question what is meant to be universal here. Is the drift-diffusion description intended as a local effective model for interactions between neighboring columns, with coefficients that should be largely independent of the global channel width? Or are the inferred parameters expected to depend on the total number of lanes and thus on the global mechanical state of the system? Related statements in the paper, such as the distinction between fast and slow fixation or the possibility of subdivision into more than two domains, are also likely to depend on system width. The broader literature on proliferating rods in confinement (see point 4 below) suggests that ordering, buckling, and stress organization can be geometry-dependent, so some explicit exploration of width dependence, even at a modest level, would substantially strengthen the paper.

2. At present, the reduction to a drift-diffusion description remains largely phenomenological. However, in this class of systems there is already a substantial body of literature connecting orientational order, its breakdown, and the underlying mechanics of confined proliferating rods. In channels and related confined geometries, nematic ordering has been linked to stress anisotropy and geometric confinement, while free and weakly confined colonies exhibit microdomain formation and (dis)ordering through the interplay of steric alignment and growth-induced extensile stresses. More recent work has further explored the role of strain-rate and subtle particle-shape effects for the stability of ordered states (see point 4). This raises the question how the effective lane-invasion dynamics described here are related to these previously identified mechanical ingredients (buckling, stresses, etc.). Even if the authors cannot address all of these points quantitatively within the present manuscript, they should at least discuss them explicitly and, ideally, probe some aspects directly.

3. I am not sure that plotting the results as a function of a length ratio is the most natural representation. For growth-rate effects, a ratio is plausible because one indeed expects relative exponential growth to be the key control parameter. For cell geometry, that is less obvious. The current x-axis of Fig. 4C suggests that only the ratio matters, but do the authors really expect the absolute initial lengths to not matter? Considering the importance of alignment, steric torques, and packing, this seems unlikely to me. Maybe it would be clearer to show the actual absolute lengths on one axis, or add explicit tests where one population is fixed at 1.0 µm and the other is varied. The inset for the neutral 1.0 µm versus 1.0 µm case is also hard to compare quantitatively with anything else (the data point for 0.4 in the main panel is already an additional "vs. 1.0µm" case, correct?). Moreover, I was surprised that the inferred drift for the nominally neutral 1.0 µm versus 1.0 µm case still appears appreciable and with relatively small error bars; I would have expected zero drift to lie within the error bars if positive and negative drifts are accounted for correctly.

4. The relevant literature is currently a bit underrepresented/narrow, both on competition in spatially structured populations and on mechanically driven ordering in proliferating bacterial systems. The manuscript would benefit from being placed more carefully in the broader context of the field, showing that competition outcomes in dense microbial populations are shaped by mechanics, confinement, and geometry across a range of systems. Some (relatively random, but all not-yet-cited) examples include Karita et al., PNAS, 2022, in confined crypt-like chambers; Postek et al., PNAS, 2024, also on drift and selection channels; Coyte et al., PNAS, 2017, on spatial structure in porous environments; Nadell et al., eLife, 2017, on spatial competition in Pseudomonas aeruginosa biofilms; Giometto et al., PNAS, 2018, on physical interactions shaping selection; van den Berg et al., Nat. Comm., 2025 on the role of nematic alignment at expanding fronts. As background for complementary systems/mechanisms, there is also Hallatschek et al., PNAS, 2007, on genetic drift and sector formation at expanding frontiers, and Farrell et al., Journal of the Royal Society Interface, 2017, on how cell shape, friction, and colony mechanics affect surfing probabilities. Effective models have also been a topic in some of these publications. Of course it is up to the authors to decide what they want to include/highlight, but if overlaps/interactions/distinctions with respect to the literature were discussed in more detail (what does the discovered mechanism add/contribute?), this would also sharpen the focus of the manuscript.

At the same time, the paper also crucially relies on mechanically induced orientational order and its destabilization in monolayers of growing spherocylinders, and that side of the field should definitely be more thoroughly discussed. Relevant are, for example, Volfson et al., PNAS, 2008 on ordering; Boyer et al., Physical Biology, 2011 on the buckling instability; You et al., Physical Review X, 2018 on the geometry and mechanics of nematic microdomains; You et al., Science Advances, 2021 on confinement-induced self-organization; Isensee et al., Journal of the Royal Society Interface, 2022 specifically on the perfect ordering in confined populations of growing rods that is the basis for this study; Langeslay and Juarez, Soft Matter, 2024 on strain-rate-controlled alignment; and Isensee and Bittihn, Soft Matter, 2025 on the sensitive dependence on particle shape. This is of course a subjective selection and some might be more relevant than others, but I think mentioning/discussing some literature more thoroughly would be very helpful to provide both background and concrete mechanistic connections (see also points 1 and 2).

5. The drift-diffusion reduction is validated only rather lightly. On the model building side, it is not shown how well the fits used to extract drift and diffusion parameters actually fit the data (and whether deviations are simply finite size effects or physically meaningful). Fixation time predictions are directly compared with theory only for one specific parameter set, while slower cases are extrapolated from fitted coefficients. That is not unreasonable given the long simulations required, but then the paper should be more careful in how strongly it states that the model “works.” There are several additional quantities that could be compared without requiring prohibitively long simulations: fixation probability within a fixed observation window, the distribution rather than only the mean of fixation times, the probability that the “wrong” population fixates when drift is small, etc. Some deeper validation would be very helpful, and if discrepancies emerge, that would actually improve the paper by clarifying the limits of the effective description.

6. The authors refer readers to previous work and supporting information for the AB model, but given that the central claims concern mechanical mechanisms, the main text should briefly state the essential ingredients: Overdamped dynamics, steric force law, friction/mobility model, etc.. Without that, it is hard to judge which aspects of the conclusions are robust and which may depend on modeling details, a question which should also at least be discussed in the manuscript (for example, is more successful lane invasion of shorter cells exclusively a geometric phenomenon, or does relative mobility also play a role? That might depend on how these mobilities are chosen.).

Minor points:

1. Line 53: since the shapes differ mainly by length rather than width, “elongated” seems more suitable than “extended.”

2. Line 81: the geometry appears inconsistent as written; the end caps seem to have a *diameter* of 1 µm, not a radius of 1 µm, if they are meant to match a 1 µm-wide rectangular body.

3. Line 146: to eliminate ambiguity, “promoting invasion *into* the bacillus population” would read more clearly.

4. While it is mentioned in the model description that cell lengths are given in terms of their initial length just after division, it would help to repeat this in the relevant figure captions, since different conventions exist in the literature.

5. Fig. 4C: the information that one length is kept constant at 2.5 µm while the other is varied is missing from the plot/caption and currently only appears in the text.

6. Fig. 4D: the caption says “size ratios,” but should probably be growth-rate ratios.

7. Throughout the manuscript, the use of the word “coccus” is somewhat unusual and potentially misleading. In the model, all particles appear to be rodlike or capsule-like objects with varying aspect ratio; the shortest ones are not actual cocci in the biological sense (which could potentially go through different geometries during elongation/division). In many places it would be clearer to write “shorter cells,” “lower-aspect-ratio cells,” or “cells with shorter initial/division length.” instead of using "coccus" as an adjective like "more coccus", etc. ("coccus-like" seems fine to me).

8. A few small typographical issues I noticed:

* line 67 there appears to be a stray quotation mark in “dynamics of‘ the latter”;

* line 77 has “less than < 1 %” is redundant;

* line 177, usage of "U", while the drift parameter is denoted by μ elsewhere;

* in the caption for Fig. S2, “Each *are* taken from 1000 simulations”.

**********

**Have the authors made all data and (if applicable) computational code underlying the findings in their manuscript fully available?**

The PLOS Data policy requires authors to make all data and code underlying the findings described in their manuscript fully available without restriction, with rare exception (please refer to the Data Availability Statement in the manuscript PDF file). The data and code should be provided as part of the manuscript or its supporting information, or deposited to a public repository. For example, in addition to summary statistics, the data points behind means, medians and variance measures should be available. If there are restrictions on publicly sharing data or code —e.g. participant privacy or use of data from a third party—those must be specified.

Reviewer #1: Yes

Reviewer #2: **No:**  There doesn't seem to be a Data Availability Statement on how to access the code etc.

**********

PLOS authors have the option to publish the peer review history of their article (what does this mean?). If published, this will include your full peer review and any attached files.

If you choose “no”, your identity will remain anonymous but your review may still be made public.

**Do you want your identity to be public for this peer review?**  For information about this choice, including consent withdrawal, please see our Privacy Policy.

Reviewer #1: No

Reviewer #2: No

**Figure resubmission:**

After uploading your figures to PLOS’s NAAS tool - https://ngplosjournals.pagemajik.ai/artanalysis, NAAS will process the files provided and display the results in the "Uploaded Files" section of the page as the processing is complete. If the uploaded figures meet our requirements (or NAAS is able to fix the files to meet our requirements), the figure will be marked as "fixed" above. If NAAS is unable to fix the files, a red "failed" label will appear above. When NAAS has confirmed that the figure files meet our requirements, please download the file via the download option, and include these NAAS processed figure files when submitting your revised manuscript.

**Reproducibility:**

To enhance the reproducibility of your results, we recommend that authors of applicable studies deposit laboratory protocols in protocols.io, where a protocol can be assigned its own identifier (DOI) such that it can be cited independently in the future. Additionally, PLOS ONE offers an option to publish peer-reviewed clinical study protocols. Read more information on sharing protocols at https://plos.org/protocols?utm_medium=editorial-email&utm_source=authorletters&utm_campaign=protocols

# Author comment: Author response to Decision Letter 1

**DOI:** 10.1371/journal.pcbi.1014874.r002

10 Jun 2026

Attachment Submitted filename: Response to Reviewers.docx

# Peer review: Decision Letter 1

**DOI:** 10.1371/journal.pcbi.1014874.r003

**Authors:** Stefan Klumpp, Tobias Bollenbach

5 Aug 2026

PCOMPBIOL-D-26-00433R1

The influence of cell morphology on the dynamics and stability of model bacterial communities

PLOS Computational Biology

Dear Dr. Milstein,

Thank you for submitting your manuscript to PLOS Computational Biology. After careful consideration, we feel that it has merit but does not fully meet PLOS Computational Biology's publication criteria as it currently stands. Therefore, we invite you to submit a revised version of the manuscript that addresses the points raised during the review process.

Please submit your revised manuscript by Oct 05 2026 11:59PM. If you will need more time than this to complete your revisions, please reply to this message or contact the journal office at ploscompbiol@plos.org. When you're ready to submit your revision, log on to https://www.editorialmanager.com/pcompbiol/ and select the 'Submissions Needing Revision' folder to locate your manuscript file.

Please include the following items when submitting your revised manuscript:

* A letter that responds to each point raised by the editor and reviewer(s). You should upload this letter as a separate file labeled 'Response to Reviewers'. This file does not need to include responses to formatting updates and technical items listed in the 'Journal Requirements' section below.

* A marked-up copy of your manuscript that highlights changes made to the original version. You should upload this as a separate file labeled 'Revised Manuscript with Track Changes'.

* An unmarked version of your revised paper without tracked changes. You should upload this as a separate file labeled 'Manuscript'.

If you would like to make changes to your financial disclosure, competing interests statement, or data availability statement, please make these updates within the submission form at the time of resubmission. Guidelines for resubmitting your figure files are available below the reviewer comments at the end of this letter.

As the corresponding author, your ORCID iD is verified in the submission system and will appear in the published article. PLOS supports the use of ORCID, and we encourage all coauthors to register for an ORCID iD and use it as well. Please encourage your coauthors to verify their ORCID iD within the submission system before final acceptance, as unverified ORCID iDs will not appear in the published article. Only the individual author can complete the verification step; PLOS staff cannot verify ORCID iDs on behalf of authors.

We look forward to receiving your revised manuscript.

Kind regards,

Stefan Klumpp

Academic Editor

PLOS Computational Biology

Tobias Bollenbach

Section Editor

PLOS Computational Biology

**Reviewers' comments:**

Reviewer's Responses to Questions

**Comments to the Authors:**

**Please note here if the review is uploaded as an attachment.**

Reviewer #1: The author has responded to all my questions and requests and. I appologize for the late submission of this recommendation, which is due to a combination of vacation, conferences, and technical issues.

Reviewer #2: Thank you very much to the authors for addressing my comments, in particular the more in-depth introduction into the field and mechanical ordering processes. The new data is very useful, but I think a few remaining issues should be addressed (they concern only the interpretation/analysis of the results):

- Thank you for adding the new 6 and 18-lanes setups. Looking at table S3, I am not sure it makes sense to call the 12-lanes and 18-lanes parameters nearly identical and the 6-lanes parameters very different. Comparing 12 and 18 lanes, the parameters are barely within error margins of each other. Also, 6 and 12 lanes is a much larger relative difference in system width than 12 and 18... Overall, based on this evidence, I would recommend the authors not to commit definitively to whether the parameters are width dependent or not (which should not be a problem since, as they point out themselves, their work is about the validity of the conceptual description, not about quantitative values).

- Related to this parameter change and possible edge effects in the 6-lanes setup, the authors indicated that "We have included a short discussion of this in the manuscript", but I couldn't find this change in the marked-up version of the manuscript (except the small comment that constant parameters seem to valid for large systems in line 149 of the marked-up version, which doesn't address/explain the new results very much).

- Similarly, for the reduced-moment-of-inertia results, has there been a mixup of numbers in Table S3? The drift seems to be identical for 12 lanes with the regular moment of inertia and I/2. The two diffusion coefficients seem to be nearly within error margins, but more importantly, the one for I/2 seems to be even *lower*, so why do the authors interpret this as a "more dynamic" boundary? Some more explanation is needed. I am sorry if I am missing something simple here... By the way, the table says L1/L2.5 while the title of Fig S6 says L1/L1.5 (different length ratio).

- I still think the inset in Fig. 4C is difficult to interpret. Its main purpose currently seems to be (now that the error bar on the drift is larger) to show that there is some diffusion but no significant drift for the L1-vs-L1 case. To make this point, it would be easier/clearer to just state the values for drift and diffusion in the text where the inset is currently referenced, especially given that this case is now included in the new L1-vs-Lx data in table S4. One could either remove the inset in Fig. 4C or modify it to show all the data from Table S4 (in any case, it would be good to show the data from table S4 also as a plot). I assume the neutral case is not shown for the Lx-vs-L2.5 data because of the long time scales involved in the L2.5-vs-L2.5 competition? Maybe it would be good to (re)state explicitly that this is why this case was only analysed in the L1-vs-Lx data when talking about neutral competition.

- The added fixation time distributions in Fig. S4 are nice, but they don't really contribute to the raised point about model validation, as long as no theory prediction from the fitted model is included. So, overall, no additional model-vs-data comparison was performed. Perhaps the authors could comment in the discussion why such comparisons beyond the mean first passage time in the inset of Fig. 5A were not done (in my naive thinking, one could just plot a theory prediction for the first passage time distribution and compare that to the distribution of the fixation times in Fig. S4, but maybe I am overlooking something), or why certain things can be expected to match the model but others not.

A minor point: I would not call the inset in Fig. 5A a "histogram of fixation times". This would imply a distribution/count of some kind (as in Fig. S4), whereas the inset in Fig. 5A really just shows fixation time averages (and the authors happened to choose a bar plot).

**********

**Have the authors made all data and (if applicable) computational code underlying the findings in their manuscript fully available?**

The PLOS Data policy requires authors to make all data and code underlying the findings described in their manuscript fully available without restriction, with rare exception (please refer to the Data Availability Statement in the manuscript PDF file). The data and code should be provided as part of the manuscript or its supporting information, or deposited to a public repository. For example, in addition to summary statistics, the data points behind means, medians and variance measures should be available. If there are restrictions on publicly sharing data or code —e.g. participant privacy or use of data from a third party—those must be specified.

Reviewer #1: Yes

Reviewer #2: Yes

**********

PLOS authors have the option to publish the peer review history of their article (what does this mean?). If published, this will include your full peer review and any attached files.

If you choose “no”, your identity will remain anonymous but your review may still be made public.

**Do you want your identity to be public for this peer review?**  For information about this choice, including consent withdrawal, please see our Privacy Policy.

Reviewer #1: No

Reviewer #2: No

[NOTE: If reviewer comments were submitted as an attachment file, they will be attached to this email and accessible via the submission site. Please log into your account, locate the manuscript record, and check for the action link "View Attachments". If this link does not appear, there are no attachment files.]

**Figure resubmission:**

**Reproducibility:**

To enhance the reproducibility of your results, we recommend that authors of applicable studies deposit laboratory protocols in protocols.io, where a protocol can be assigned its own identifier (DOI) such that it can be cited independently in the future. Additionally, PLOS ONE offers an option to publish peer-reviewed clinical study protocols. Read more information on sharing protocols at https://plos.org/protocols?utm_medium=editorial-email&utm_source=authorletters&utm_campaign=protocols

# Author comment: Author response to Decision Letter 2

**DOI:** 10.1371/journal.pcbi.1014874.r004

18 Sep 2026

Attachment Submitted filename: Response to Reviewers II.pdf

# Peer review: Decision Letter 2

**DOI:** 10.1371/journal.pcbi.1014874.r005

**Authors:** Stefan Klumpp, Tobias Bollenbach

4 Oct 2026

Dear Dr. Milstein,

We are pleased to inform you that your manuscript 'The influence of cell morphology on the dynamics and stability of model bacterial communities' has been provisionally accepted for publication in PLOS Computational Biology.

Before your manuscript can be formally accepted you will need to complete some formatting changes, which you will receive in a follow up email. A member of our team will be in touch with a set of requests.

Please note that your manuscript will not be scheduled for publication until you have made the required changes, so a swift response is appreciated.

IMPORTANT: The editorial review process is now complete. PLOS will only permit corrections to spelling, formatting or significant scientific errors from this point onwards. Requests for major changes, or any which affect the scientific understanding of your work, will cause delays to the publication date of your manuscript.

Should you, your institution's press office or the journal office choose to press release your paper, you will automatically be opted out of early publication. We ask that you notify us now if you or your institution is planning to press release the article. All press must be co-ordinated with PLOS.

Thank you again for supporting Open Access publishing; we are looking forward to publishing your work in PLOS Computational Biology.

Best regards,

Stefan Klumpp

Academic Editor

PLOS Computational Biology

Tobias Bollenbach

Section Editor

PLOS Computational Biology

***********************************************************

Reviewer's Responses to Questions

**Comments to the Authors:**

**Please note here if the review is uploaded as an attachment.**

Reviewer #2: Thank you very much to the authors for addressing all my comments satisfactorily. I am happy to support publication.

**********

**Have the authors made all data and (if applicable) computational code underlying the findings in their manuscript fully available?**

The PLOS Data policy requires authors to make all data and code underlying the findings described in their manuscript fully available without restriction, with rare exception (please refer to the Data Availability Statement in the manuscript PDF file). The data and code should be provided as part of the manuscript or its supporting information, or deposited to a public repository. For example, in addition to summary statistics, the data points behind means, medians and variance measures should be available. If there are restrictions on publicly sharing data or code —e.g. participant privacy or use of data from a third party—those must be specified.

Reviewer #2: Yes

**********

PLOS authors have the option to publish the peer review history of their article (what does this mean?). If published, this will include your full peer review and any attached files.

If you choose “no”, your identity will remain anonymous but your review may still be made public.

**Do you want your identity to be public for this peer review?**  For information about this choice, including consent withdrawal, please see our Privacy Policy.

Reviewer #2: No

# Editor report: Acceptance letter

**DOI:** 10.1371/journal.pcbi.1014874.r006

**Authors:** Stefan Klumpp, Tobias Bollenbach

PCOMPBIOL-D-26-00433R2

The influence of cell morphology on the dynamics and stability of model bacterial communities

Dear Dr Milstein,

I am pleased to inform you that your manuscript has been formally accepted for publication in PLOS Computational Biology. Your manuscript is now with our production department and you will be notified of the publication date in due course.

The corresponding author will soon be receiving a typeset proof for review, to ensure errors have not been introduced during production. Please review the PDF proof of your manuscript carefully, as this is the last chance to correct any errors. Please note that major changes, or those which affect the scientific understanding of the work, will likely cause delays to the publication date of your manuscript.

Soon after your final files are uploaded, unless you have opted out, the early version of your manuscript will be published online. The date of the early version will be your article's publication date. The final article will be published to the same URL, and all versions of the paper will be accessible to readers.

For Research, Software, and Methods articles, you will receive an invoice from PLOS for your publication fee after your manuscript has reached the completed accept phase. If you receive an email requesting payment before acceptance or for any other service, this may be a phishing scheme. Learn how to identify phishing emails and protect your accounts at https://explore.plos.org/phishing.

Thank you again for supporting PLOS Computational Biology and open-access publishing. We are looking forward to publishing your work!

With kind regards,

Sharmila Kamatchi

PLOS Computational Biology | Carlyle House, Carlyle Road, Cambridge CB4 3DN | United Kingdom ploscompbiol@plos.org | Phone +44 (0) 1223-442824 | ploscompbiol.org | @PLOSCompBiol
